# A Rare Case of Facial Arteriovenous Malformation in a 3‐Year‐Old Female

**DOI:** 10.1002/ccr3.70155

**Published:** 2025-01-27

**Authors:** Bernadette Pedun, Ivaan Pitua, Felix Bongomin, Valeria Nabbosa, Samuel Bugeza

**Affiliations:** ^1^ Uganda Cancer Institute Kampala Uganda; ^2^ School of Medicine, College of Health Sciences Makerere University Kampala Uganda; ^3^ Department of Internal Medicine Gulu Regional Referral Hospital Gulu Uganda; ^4^ Department of Medical Microbiology and Immunology Gulu University Gulu Uganda; ^5^ Department of Radiology and Radiotherapy, School of Medicine, College of Health Sciences Makerere University Kampala Uganda

**Keywords:** arteriovenous malformation, case report, computed tomography angiography, maximum intensity projection, pediatrics

## Abstract

Arteriovenous malformation (AVM) is a rare congenital vascular anomaly involving abnormal artery–vein connections that bypass the capillary system. AVMs are particularly uncommon in young children. A 3‐year‐old girl presented with a painless, progressively enlarging left cheek swelling since birth. Clinical examination showed left facial asymmetry with a nontender, pulsatile mass in the left parotid region without skin changes or visible collateral vessels. Computed tomography angiography revealed an enhancing mass in the left parotid region with feeding and draining vessels. The patient was referred to vascular surgery for further management. AVM's rarity in pediatrics highlights the need for further research in genetic and developmental factors, aiming to improve early detection, management, and outcomes for affected children.

**Summary**

- This case of arteriovenous malformation in a 3‐year‐old girl presenting as a painless facial swelling highlights the role of imaging for early diagnosis
- Arteriovenous malformation in pediatric patients poses specific risks, making early detection and referral critical for improving the management options and reducing any potential complications in young children.

## 1 Introduction

Arteriovenous malformation (AVM) is a rare vascular abnormality involving direct connections between arteries and veins, bypassing the capillary network [1, 2, 3]. AVMs should be differentiated from vascular tumors, which have distinct clinical courses and outcomes. Notably, about 90% of AVMs regress spontaneously by age 9 [4, 5], with an estimated prevalence of 5 in 10,000 people globally [6].

AVMs are congenital and may present at any age, although diagnosis in young children is rare, with a mean age of 31.2 years [3]. Females are four times more likely to be diagnosed with AVMs than males, and these lesions are more common in Caucasians [4]. Although the exact etiology is often idiopathic, AVMs are thought to result from abnormal vascular development during the third week of embryonic life, possibly due to mutations in tyrosine kinase receptors and G‐protein signaling pathways [2, 7]. Associations with low birth weight, multiple gestations, and chorionic villus sampling have also been noted [4].

Although AVMs can occur anywhere, 60% appear in the head and neck region in children [4, 6]. Extracranial AVMs in the neck may be asymptomatic and found incidentally, or they may present as birthmarks, painless lumps, or enlarging swellings [2, 4, 8]. Due to their proximity to major blood vessels and nerves, neck AVMs pose significant hemorrhage risk and warrant careful assessment.

Radiologic imaging is essential for diagnosing AVMs. Ultrasound, computed tomography angiography (CTA), and magnetic resonance angiography (MRA) are commonly used to assess the vascular anomaly, its extent, and its relationship to nearby structures [8, 9, 10]. CTA provides detailed visualization of feeding arteries, draining veins, and the AVM nidus, whereas MRA offers a noninvasive option to evaluate vascular flow dynamics and differentiate AVMs from other lesions [1, 5, 10, 11]. Herein, we report in details, the clinical presentation and CT angiographic findings of a facial AVM in a Ugandan child.

## 2 Case Presentation

A 3‐year‐old female was brought to the imaging department with a history of a progressively enlarging swelling of the left cheek since birth. The parents reported that the swelling had been present as a small, soft lump at birth, but over the past 2 years, it had gradually increased in size, becoming more prominent though the child usually did not exhibit irritability when the area was touched. There were no reports of any bump elsewhere in the body nor redness, warmth, or tenderness in the area, and the child's general health was otherwise normal, with no significant medical history or developmental delays.

Clinical examination revealed a nontender, pulsatile mass measuring approximately 6 × 8 cm in the left preauricular region. The overlying skin appeared normal, without any signs of discoloration or visible veins. Palpation of the mass revealed a firm consistency and was pulsatile, suggesting a possible vascular origin. There was no regional lymphadenopathy noted.

**FIGURE 1:**
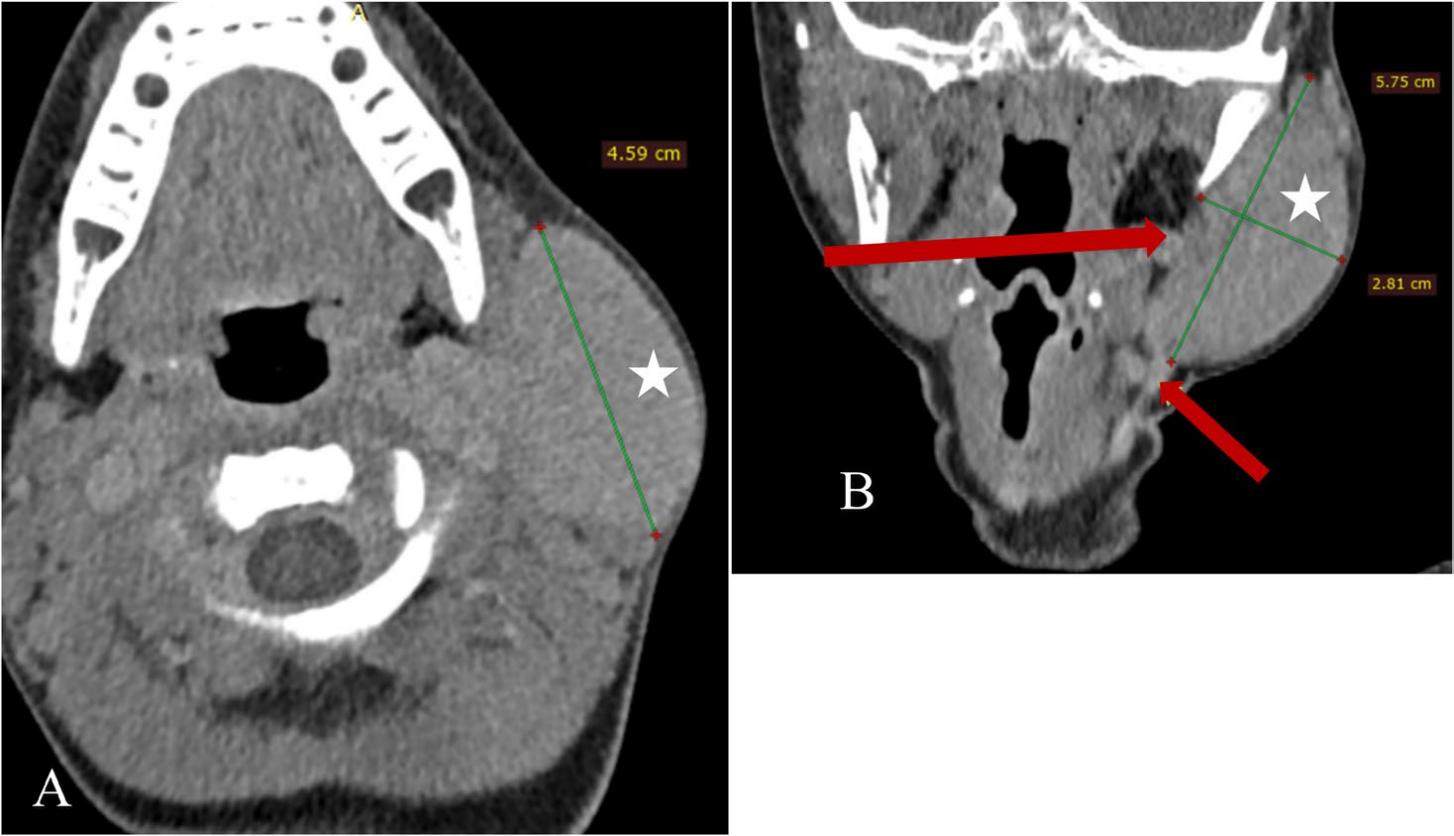
(A) and (B) are axial noncontrast CT of head and neck with a coronal reformat, respectively, showing an expansile soft tissue density well‐marginated mass (white star) in the left parotid region, which is nondestructive to the adjacent bone and does not breach the skin. It measures 5.75 × 4.59 × 2.81 cm. Red arrows show blood vessels.

**FIGURE 2:**
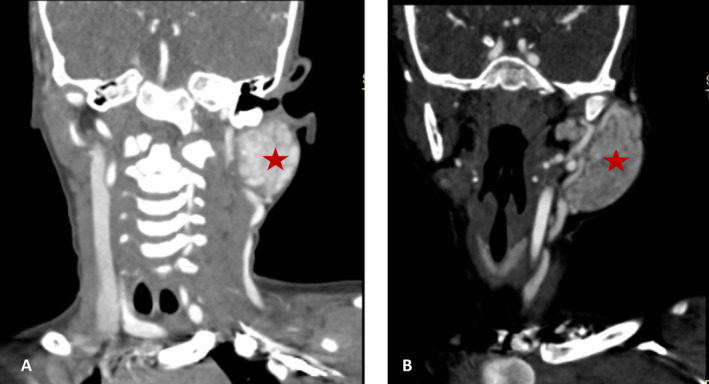
(A) and (B) are contrasted head and neck CT coronal reformats with the mass showing avid enhancement in the arterial phase, with retention of contrast in the venous phase, respectively. This makes this mass (red star) an arteriovenous malformation.

Diagnostic imaging by CTA was performed under general anesthesia, utilizing reformats and maximum intensity projection (MIP) to visualize the lesion's vascular architecture. The imaging revealed a mass, measuring 5.75 × 4.59 × 2.81 cm, which appeared as an expansile, well‐marginated soft tissue density within the left parotid region (Figure 1), with no destruction of adjacent bone structures and no skin breach. The imaging also showed a hypervascular mass in the left parotid region, which exhibited early and intense contrast enhancement in the arterial phase (Figure 2).

**FIGURE 3:**
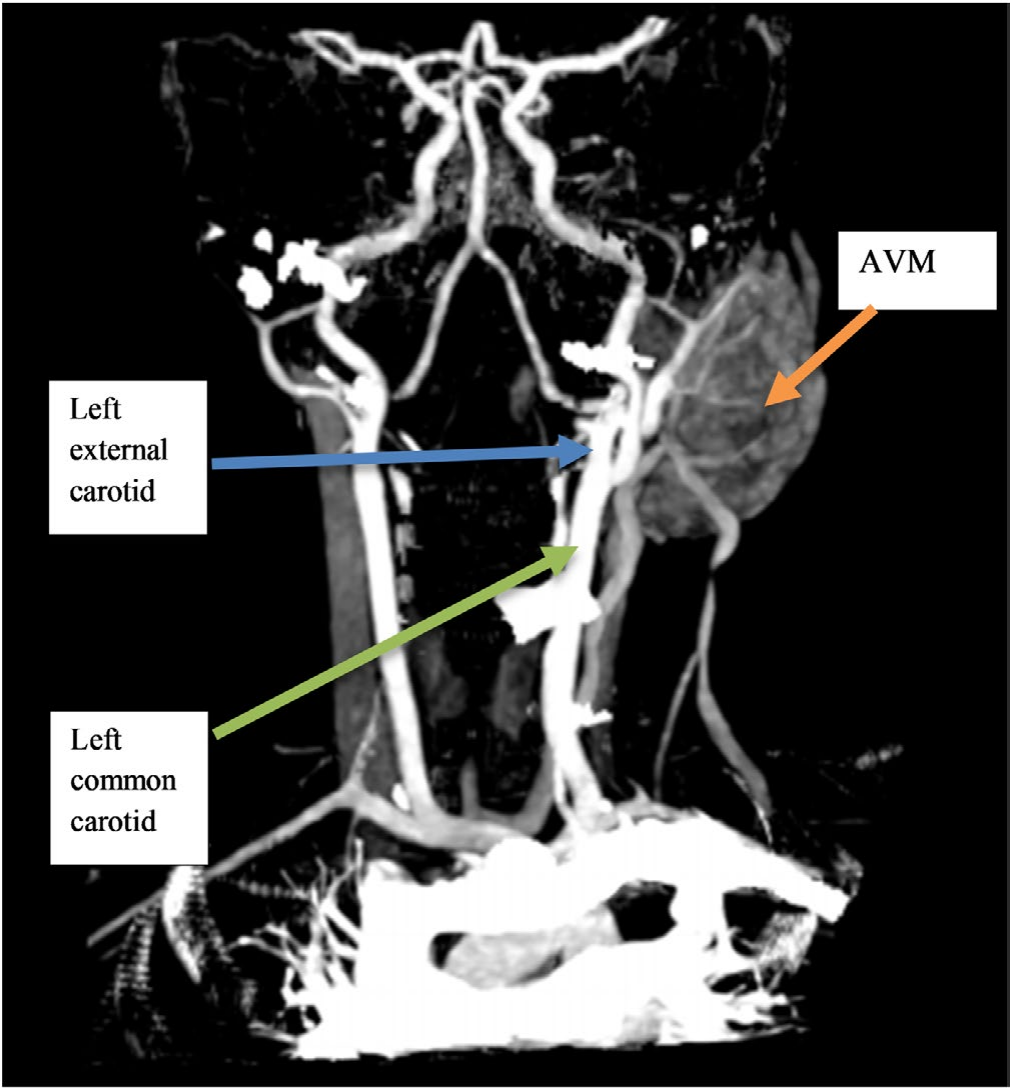
A maximum intensity projection of the head and neck vessels showing a mixed density mass in the left parotid region, with a nodular, tortuosity of the intertwining arteries and veins giving it the characteristic “bag of worms” appearance. A feeding vessel, which is the left posterior auricular artery, is noted branching into the mass, with another feeder seen from the left subclavian artery. There are multiple draining veins noted with some separated into a hypoplastic and inferiorly duplicated left external jugular.

**FIGURE 4:**
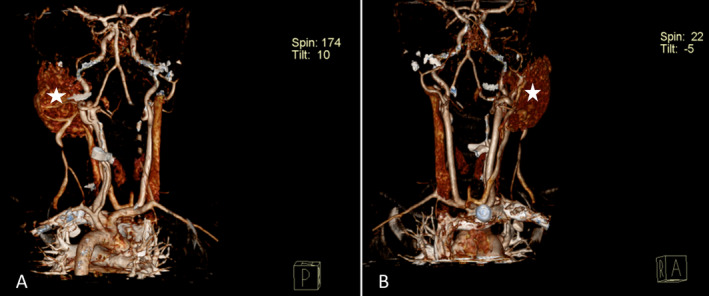
(A) and (B) are 3D reconstructions, posterior and anterior views, respectively. These show multiple intertwining vessels (white star) in the left parotid region of the patient, with a similar appearance to both arteries and veins.

Notably, a prominent feeding artery and draining vein were observed (Figure 1), indicative of a high blood flow within the lesion. Additionally, MIP imaging highlighted the intricate vascular network, revealing a tortuous collection of intertwining arteries and veins, giving the mass a “bag of worms” appearance typical of AVM (Figure 3). The primary feeding vessels, originating from the left posterior auricular artery and left subclavian artery, supplied the mass, with multiple draining veins observed, including a hypoplastic and inferiorly duplicated left external jugular vein (Figure 3). Three‐dimensional reconstructions further detailed the arrangement of these vessels, showing the distinctive intertwining structure of arteries and veins in the parotid region (Figure 4).

On the basis of these findings, a diagnosis of an AVM in the left parotid region was confirmed, and the child was referred to vascular surgeons for further management, although intervention had not been undertaken at the time of writing this case report.

## 3 Differential Diagnosis

Our first differential was a hemangioma, which is a benign vascular tumor, often present similar to soft, compressible masses but differ in their spontaneous regression over time and characteristic enhancement patterns on imaging. Another consideration was a lymphatic malformation, such as a cystic hygroma, typically presenting with a soft, nonpulsatile mass that is less likely to show the rapid blood flow associated with AVMs. Additionally, soft tissue sarcomas, though less common in young children, was a consideration to be ruled out, especially because there were no signs of tissue invasion or rapid growth. Carotid body tumors, although rare in pediatric cases, may present with a similar facial mass, but these tumors are usually located along the carotid artery bifurcation and have distinct imaging features. In this case, the presence of prominent feeding and draining vessels on CTA with a characteristic “bag of worms” appearance confirmed the diagnosis of an AVM.

## 4 Discussion

AVM in very early childhood is an uncommon and rare occurrence [3]. There was no known cause of the condition in the case discussed here and it tallies with literature elsewhere [1, 4]. The patient also did not have any known risk factors such as prematurity, low birth weight, similar family history, or genetic history of vascular malformations as is the case with some literature [4]. Some studies strongly associate the female fetus to have a higher chance of developing AVMs, and because the case discussed is also a female, it is in agreement with that literature [4]. Some literature suggests that AVMs are very stable or indolent masses, growing with hormonal influence of the growth spurts, especially in adolescence [1, 5, 8], although this was not consistent with the presentation of our patient in whom there was a progressive increase in size from a few months after birth.

In young children, AVMs often manifest with subtle and nonspecific symptoms, making early detection difficult [4]. The lack of communication abilities in toddlers can further hinder the identification of neurological deficits, especially for cerebral AVMs, delaying the diagnosis and intervention. In this case, the very young nonverbal age of the patient, the nontender nature of the swelling, and absence of discoloration or visible veins on physical examination made it challenging to immediately attribute the mass to a vascular origin.

Diagnostic imaging techniques, particularly ultrasound, magnetic resonance imaging (MRI), and CTA, play an important role in confirming the presence of AVMs and understanding their vascular architecture [8, 10] to distinguish them from other vascular anomalies such as vascular tumors. In pediatric cases, the use of radiation‐free imaging methods, such as ultrasound and MRI, is particularly advantageous. Doppler ultrasound is invaluable for initial evaluation, providing real‐time assessment of blood flow patterns and helping distinguish AVMs from other vascular anomalies [5]. MRI, and specifically MRA, offers a detailed visualization of the vascular architecture and flow dynamics without ionizing radiation. These modalities are especially crucial in pediatric patients where cumulative radiation exposure should be minimized, and their use should be prioritized whenever possible [5]. AVMs typically exhibit distinct CTA characteristics that aid in their identification and classification [11]. The CTA characteristics of AVMs include rapid and intense contrast enhancement due to the presence of direct arteriovenous shunting [5, 11]. This enhancement occurs in both the arterial and venous phases, contributing to the “early filling and rapid washout” pattern. The CT images often reveal dilated feeding arteries supplying the AVM and enlarged draining veins, creating a characteristic “bag of worms” appearance [3, 5, 11]. In this case, CTA was done but no prior ultrasonography was reported to have been performed to obtain the spectral Doppler characteristics. The imaging revealed a well‐marginated mass in the left parotid region, which was nondestructive to the adjacent bone and did not breach the skin, and was avidly enhancing and rapidly washing out in venous phase. Three‐dimensional reformats showed a tangled network of enlarged and tortuous vessels as described by prior literature consistent with an AVM diagnosis.

The therapeutic considerations for AVMs in pediatric patients are multifaceted and management is a multidisciplinary approach. Given the patient's young age and the extensive nature of the AVM, endovascular embolization in advanced settings would be chosen as a preliminary step to reduce the blood flow within the lesion and decrease its size [7, 12]. The partial embolization successfully results in a reduction in the size of the AVM, demonstrating the potential benefits of such interventions in young children [6, 8]. However, the management of AVMs in pediatric patients should be approached with caution due to the potential risks and challenges involved. The patient discussed here was yet to get a clear management plan but was appropriately referred to the pediatric vascular surgeons for management.

Long‐term follow‐up and regular imaging are imperative to monitor the AVM's progression and evaluate the need for further interventions or surgical resection in the future. Close monitoring will also help detect any potential neurological deficits or developmental delays associated with the AVM. Potential complications from either the AVMs or the intervention itself may include spontaneous rupture and bleeding or hematoma collection at the site, puncture site hematoma, retrograde embolization of normal arteries, bronchospasm, pulmonary edema, and pulmonary embolism [3, 6, 8]. None of these complications were seen in the case discussed here but going forward, should be carefully evaluated.

## 5 Conclusion and Results (Outcome and Follow‐Up)

This case of an AVM in the left parotid region of a 3‐year‐old child showed the importance of imaging in accurately diagnosing vascular malformations in pediatric patients with facial masses. The CT imaging was performed using a three‐phase protocol to comprehensively evaluate the vascular architecture of the lesion. Standard soft tissue, bone, and vascular windows were utilized to ensure detailed visualization of the mass, its relationship with adjacent structures, and the vascular supply. CTA findings, including early and intense contrast enhancement, multiple feeding arteries, and draining veins, were critical in confirming the diagnosis and defining the lesion's vascular architecture. The patient was referred to a vascular surgery team for potential endovascular embolization, although no intervention had been undertaken at the time of writing. Long‐term follow‐up will be essential to monitor the lesion's progression, manage symptoms, and address potential complications. This case emphasizes that early detection and multidisciplinary management are key to optimizing outcomes for pediatric patients with AVMs, as these lesions carry risks of hemorrhage and other complications if left untreated.

## Author Contributions

**Bernadette Pedun:** conceptualization, data curation, formal analysis, investigation, methodology, project administration, resources, software, supervision, validation, writing – original draft, writing – review and editing. **Ivaan Pitua:** methodology, software, validation, writing – original draft, writing – review and editing. **Felix Bongomin:** methodology, supervision, writing – original draft, writing – review and editing. **Valeria Nabbosa:** methodology, writing – original draft, writing – review and editing. **Samuel Bugeza:** investigation, methodology, writing – original draft, writing – review and editing.

## Ethics Statement

No institutional approval was required to publish the case details. The patient's parent provided an informed written consent to participate in the study.

## Consent

Written informed consent for publication of this case report and accompanying images was obtained from the patient's parent.

## Conflicts of Interest

The authors declare no conflicts of interest.

## Data Availability

All relevant data and materials are available within the text.
